# Tailoring the magnetic anisotropy of Py/Ni bilayer films using well aligned atomic steps on Cu(001)

**DOI:** 10.1038/srep11055

**Published:** 2015-06-11

**Authors:** S. Ma, A. Tan, J. X. Deng, J. Li, Z. D. Zhang, C. Hwang, Z. Q. Qiu

**Affiliations:** 1 Shenyang National Laboratory for Materials Science, Institute of Metal Research, Chinese Academy of Sciences, Shenyang 110016, China; 2 Department of Physics, University of California at Berkeley, Berkeley,California 94720; 3 Korea Research Institute of Standards and Science, Yuseong, Daejeon 305-340, Korea

## Abstract

Tailoring the spin orientation at the atomic scale has been a key task in spintronics technology. While controlling the out-of-plane to in-plane spin orientation has been achieved by a precise control of the perpendicular magnetic anisotropy at atomic layer thickness level, a design and control of the in-plane magnetic anisotropy has not yet been well developed. On well aligned atomic steps of a 6° vicinal Cu(001) surface with steps parallel to the [110] axis, we grow Py/Ni overlayer films epitaxially to permit a systematic exploration of the step-induced in-plane magnetic anisotropy as a function of both the Py and the Ni film thicknesses. We found that the atomic steps from the vicinal Cu(001) induce an in-plane uniaxial magnetic anisotropy that favors both Py and Ni magnetizations perpendicular to the steps, opposite to the behavior of Co on vicinal Cu(001). In addition, thickness-dependent study shows that the Ni films exhibit different magnetic anisotropy below and above ~6 ML Ni thickness.

Engineering the spin orientation at the atomic scale in a magnetic nanostructure has been one of the most important issues in spintronics development. Since the spin orientation in a ferromagnetic crystal is determined by the so-called magnetic anisotropy^1^,^2^,^3^, a design and control of the magnetic anisotropy by atomic engineering has been one of the most challenging topics in nanomagnetism research^4^,^5^,^6^,^7^.

For out-of-plane to in-plane spin orientation of a magnetic thin film, great success has been achieved by tailoring the perpendicular magnetic anisotropy by controlling the film thickness at atomic layer level^8^,^9^. Moreover, many important and interesting phenomena have been discovered such as the stripe^10^,^11^,^12^ and bubble phases in continuous films^13^, and the topological magnetic skyrmions in patterned films^14^. In comparison, limited success has been achieved in understanding and tailoring the in-plane spin orientation of a ferromagnetic thin film. In an attempt to engineer the in-plane magnetic anisotropy, atomic steps on vicinal films have been employed to induce in-plane uniaxial magnetic anisotropy^15^,^16^,^17^,^18^,^19^,^20^,^21^. The general approach to the control of the in-plane magnetic anisotropy has been more or less limited to the modulation of the vicinal surface by various methods. For example, it was shown recently that a direct heating of a vicinal Si(111) can effectively modulate the magnetic anisotropy of an Fe overlayer^6^, and that microstep- and macrostep-bunched vicinal Si(111) substrates could modulate the Co overlayer magnetic anisotropy very differently^22^. However, it is unclear on how the vicinal surface exactly modulates the magnetic anisotropy at different film thicknesses^7^,^22^.

Noticing that the d electrons in transition metals are very sensitive to their local environment and sometimes even exhibit exotic anisotropy behaviors such as the quantum size effect^23^,^24^,^25^, it is very important and constructive to design a well defined system whose anisotropy can be modulated continually in both the out-of-plane and the in-plane directions as a function of the ferromagnetic film thickness. For this purpose, we fabricated Py/Ni bilayer films on vicinal Cu(001) surface. Ni on Cu(001) has a tetragonal distorted fct structure which carries a volume-type out-of-plane uniaxial anisotropy to result in an in-plane to out-of-plane spin reorientation transition (SRT) with increasing the Ni film thickness^26^. However, the step-induced in-plane magnetic anisotropy cannot be fully studied because of the limited thickness range below the Ni SRT thickness. For example, Dhesi *et al.* investigated the SRT of Ni film on vicinal Cu(1 1 32) and found a very peculiar behavior that below the SRT thickness, the Ni magnetization is titled from the surface normal direction with the in-plane component perpendicular to the steps above 5.5 ML and then switches to in-plane direction and parallel to the steps below 5.5 ML^27^. Thus it has remained as a mystery on the sign of the step-induced magnetic anisotropy in vicinal Ni/Cu(001): is the easy axis parallel to the steps as in the Co/vicinal Cu(001) or perpendicular to the steps?

In this work, we studied Py/Ni bilayer films on vicinal Cu(001) to permit a modulation of the magnetic anisotropy in both the out-of-plane and the in-plane directions. We found that both Py and Ni films on vicinal Cu(001) have a step-induced in-plane magnetic anisotropy that favors the magnetization perpendicular to the steps, which is opposite to the result in vicinal Co/Cu(001) system. Moreover, the step-induced anisotropy has different values below and above ~5–6 ML Ni thickness. Finally, systematic thickness-dependent study allows us to separate the volume and surface contributions to the step-induced magnetic anisotropy. Our result provides a new method for the modulation of the magnetic anisotropy in both the out-of-plane and the in-plane directions which is important to the prospective application in future spintronics devices.

## Results

### Spin reorientation transition (SRT)

To obtain the Ni SRT thickness as a function of Py thickness, both polar and longitudinal (field perpendicular to the steps) hysteresis loops were measured on Cu/Py/Ni/Cu(001) by scanning the MOKE laser along the Ni wedge at a fixed Py thickness. After finishing a one line scan, the laser beam position was moved to a new position along the Py wedge to change the Py thickness. Then polar and longitudinal MOKE loops were taken again by scanning the MOKE laser beam along the Ni wedge.

**Figure 1:**
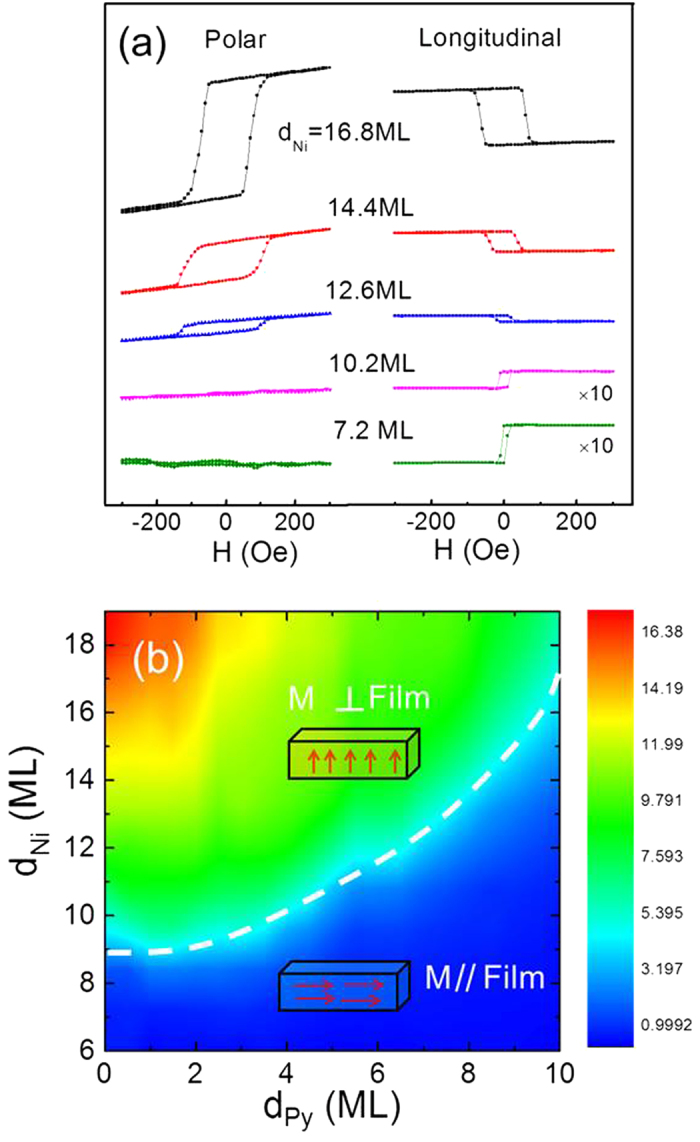
(**a**) Polar and longitudinal hysteresis loops of vicinal Cu/Py(6 ML)/Ni/Cu(001) show clearly the SRT from in-plane to out-of-plane directions as the Ni film increases above 11 ML. The in-plane magnetic field was applied perpendicular to the atomic steps for longitudinal loop measurement. (**b**) Polar MOKE signal (arb. unit) in the Py-Ni thickness plane. Dashed line indicates the SRT point.

Figure 1(a) shows typical polar and longitudinal hysteresis loops of a line scan along the Ni wedge at 6 ML Py thickness (d_Py_ = 6 ML). Below 11 ML Ni thickness (d_Ni_ < 11 ML), no polar hysteresis loops were observed. In contrast, the longitudinal MOKE exhibits a square shape hysteresis loop, showing that the Cu/Py(6 ML)/Ni/Cu(001) film has an in-plane magnetization for d_Ni_ < 11 ML. Above 11 ML Ni thickness, the polar hysteresis loop develops into a square shape with increasing Ni thickness. Meanwhile, the longitudinal hysteresis loop reverses direction with a much greater magnitude. This behavior indicates a SRT of the Py/Ni magnetization from in-plane to out-of-plane direction as Ni film thickness increases above 11 ML. The reversed longitudinal MOKE loop with a much greater magnitude is due to a polar MOKE signal in the vicinity of the SRT, which was already reported previously in an Fe/Ag(001) system^9^. The result of Fig. 1(a) shows that Cu/Py(6 ML)/Ni/Cu(001) undergoes a SRT at d_Ni_ ≈ 11 ML, which is thicker than the Cu/Ni/Cu(001) SRT thickness of d_Ni_ ≈ 10 ML. This is because Py/Cu(001) has an in-plane magnetization so that once the Py and Ni are coupled together the Ni perpendicular magnetic anisotropy in Py/Ni/Cu(001) has to overcome an extra Py in-plane shape anisotropy in order to give a perpendicular magnetization.

To have a systematic thickness-dependent study of the SRT, we plotted the polar remanence of the Py/Ni films in the d_Py_-d_Ni_ thickness plane [Fig. 1(b)]. The SRT character shown in Fig. 1(b) exists for all Py thicknesses studied: the polar MOKE signal develops above a critical Ni thickness as a result of in-plane to out-of-plane SRT. We define a ~5% polar MOKE signal as the practical definition of the SRT point [dashed line in Fig. 1(b)]. Then it is clearly seen that the Ni SRT thickness increases with increasing Py thickness. Similar behavior was also observed in a Co/Ni/Cu(001) system^28^.

### Step-induced in-plane uniaxial magnetic anisotropy in vicinal Cu/Py/Cu(001)

**Figure 2:**
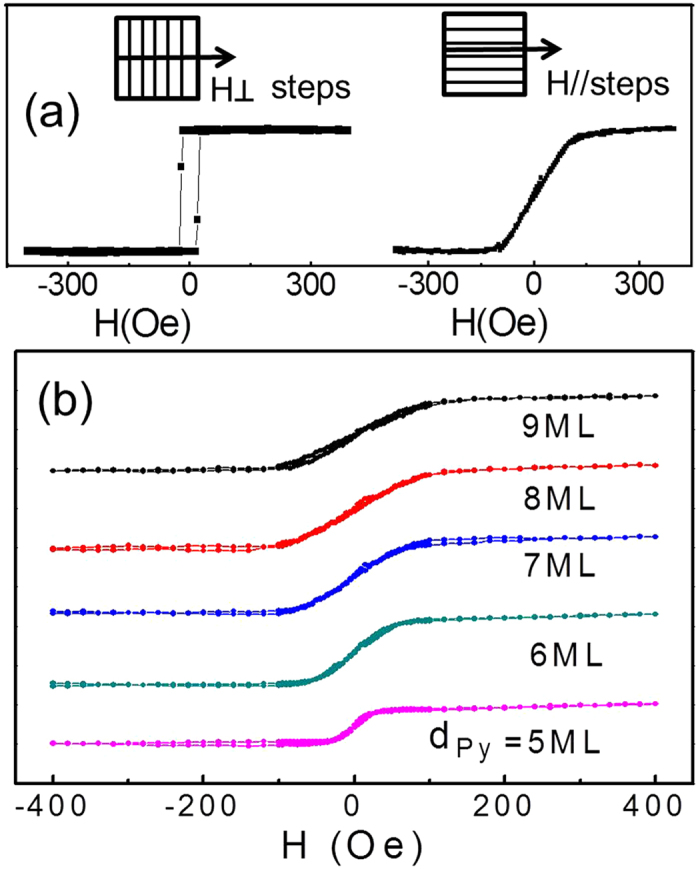
(**a**) Longitudinal hysteresis loops of vicinal Cu/Py(10 ML)/Cu(001) show the existence of a step-induced uniaxial magnetic anisotropy with the easy magnetization axis perpendicular to the atomic steps. (**b**) The hard axis saturation field increases with increasing the Py thickness.

To study the step-induced magnetic anisotropy, we first performed longitudinal MOKE measurements on vicinal Cu/Py(10 ML)/Cu(001) film. For magnetic field perpendicular to the atomic steps, a square shape hysteresis loop was observed with a full remanence. For the field parallel to the atomic steps, the film exhibited a hard axis hysteresis loop with zero remanence [Fig. 2(a)]. The above observation shows that the atomic steps on the vicinal Cu(001) substrate induce a uniaxial magnetic anisotropy in the Py overlayer with the easy magnetization axis perpendicular to the atomic steps. This result is opposite to the vicinal Co/Cu(001) system where the step-induced uniaxial magnetic anisotropy favors the easy magnetization axis parallel to the atomic steps. We then measured the longitudinal MOKE at different Py thicknesses by applying the magnetic field parallel to the step direction. The vicinal Cu/Py/Cu(001) always exhibited the hard axis loops [Fig. 2(b)]. Moreover, we found that the saturation field increases with increasing Py thickness. Note that the saturation field is a measure of the strength of the step-induced magnetic anisotropy, so the increase of the hard axis loop saturation field shows that the step-induced magnetic anisotropy increases with increasing Py film thickness. To understand this behavior, we need to determine quantitatively the surface and volume contribution of the step-induced anisotropies.

**Figure 3:**
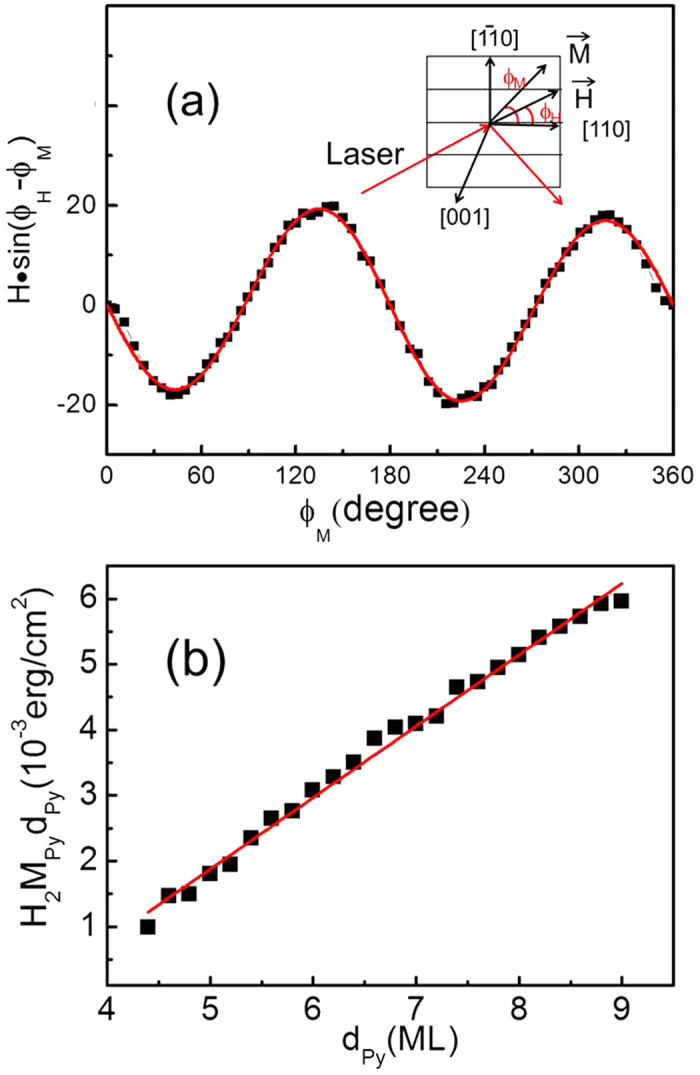
(**a**) Magnetic torque of Hsin(*ϕ*_*H*_ − *ϕ*_*M*_)vs *ϕ*_*M*_ from ROTMOKE measurement on vicinal Cu/Py(6 ML)/Cu(001). The red line is the fitting result using eqn. (2). (**b**) The anisotropy field versus Py thickness. A linear fit using eqn. (3) determines the surface and volume parts of the step-induced uniaxial magnetic anisotropy.

To obtain the magnetic anisotropy quantitatively, we performed ROTMOKE measurements on the sample. The in-plane projection of the incident laser beam was set to be parallel to the in-plane hard magnetization axis (parallel to the steps). As the magnetic field *H* = 200 Oe rotated in the film plane, the ROTMOKE signal picked up the projection of the magnetization along the hard axis [inset of Fig. 3(a)]. Then the angular difference between the magnetic field and the magnetization contains the information of the magnetic anisotropy. Since the Py 4-fold anisotropy is negligibly small, the magnetic anisotropy energy per unit area can be written as


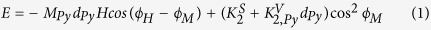


Here *H* = 200 Oe is the strength of the rotating magnetic field, *ϕ*_*H*_ and *ϕ*_*M*_ are the angles of the magnetic field and magnetization with respect to the atomic steps [inset of Fig. 3(a)], 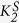 and 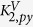 are the surface (including both Py/Cu interfaces) and volume parts of the step-induced uniaxial magnetic anisotropy, respectively. Minimizing eqn. (1) yields the magnetic torque equilibrium equation.


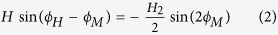



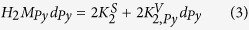


ROTMOKE measures cos*ϕ*_*M*_ as a function of *ϕ*_*H*_ (5^o^ per step) from which we determine *ϕ*_*M*_. Then the torque of *H* sin(*ϕ*_*H*_ − *ϕ*_*M*_) can be determined in the experiment as a function of *ϕ*_*M*_ [Fig. 3(a)]. Fitting the experimental data using eqn. (2) yields the red line in Fig. 3(a), which fits the data very well. We repeated the ROTMOKE measurement as a function of Py thickness and plotted the corresponding *H*_*2*_*M*_*Py*_*d*_*Py*_ in Fig. 3(b), where *M*_*Py*_ = 800 Oe and 1.8 Å/ML have been taken for Py. The result shows a pretty good linear dependence as given by eqn. (3). Then fitting the data in Fig. 3(b) yields 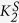 = −(1.8 ± 0.1) × 10^−3^ erg/cm^2^ and 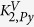 = (3.1 ± 0.1) × 10^4^ erg/cm^3^ for the surface and volume parts of the step-induced magnetic anisotropy.

### Step-induced in-plane uniaxial magnetic anisotropy in vicinal Cu/Py/Ni/Cu(001)

**Figure 4:**
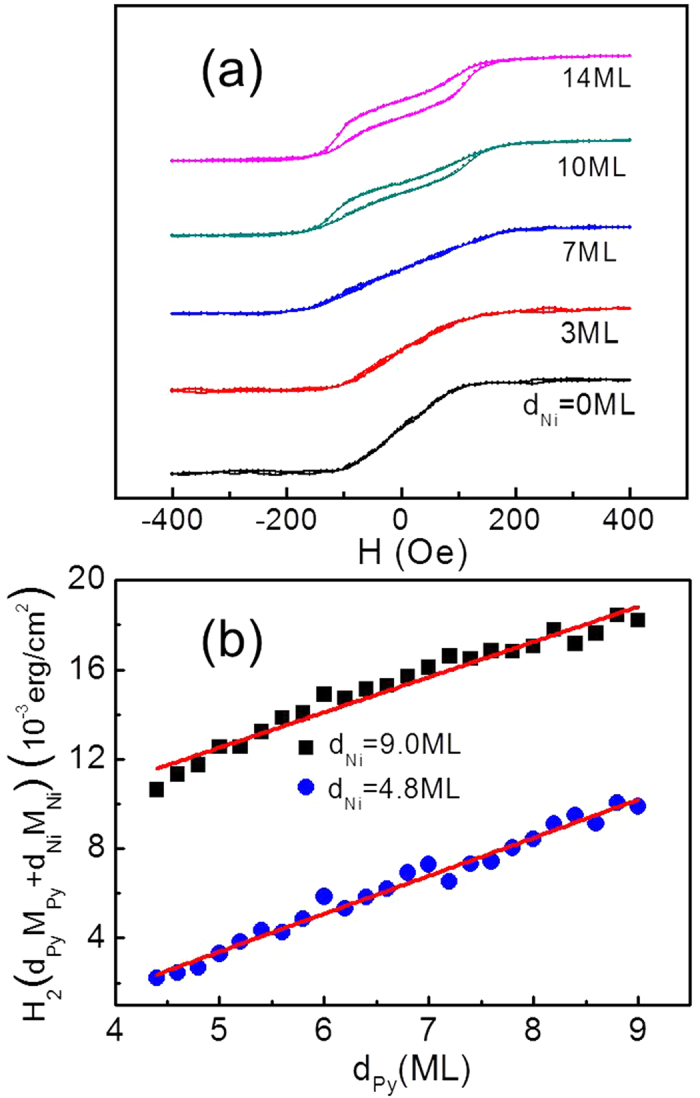
(**a**) Hard axis (H parallel to the steps) longitudinal hysteresis loops from vicinal Cu/Py(10 ML)/Ni/Cu(001). The Ni film enhances the step-induced magnetic anisotropy. (**b**) Anisotropy field versus Py thickness for vicinal Cu/Py/Ni(4.8 ML)/Cu(001) and Cu/Py/Ni(9.0 ML)/Cu(001). The same slope of the two curves shows that the Py volume anisotropy (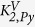) remains the same for 4.8 ML<d_Ni_ < 9.0 ML.

After studying the step-induced anisotropy in vicinal Cu/Py/Cu(001) films, we studied the step-induced anisotropy in vicinal Cu/Py/Ni/Cu(001) films. We first measured the longitudinal MOKE hysteresis loops of vicinal Cu/Py(10 ML)/Ni/Cu(001) for the field along the hard axis (parallel to the steps). The saturation field increases with increasing Ni thickness, showing that the Ni film enhances the step-induced uniaxial magnetic anisotropy [Fig. 4(a)]. In other words, there should exist a step-induced uniaxial anisotropy in the Ni film which also favors the easy magnetization axis perpendicular to the atomic steps, the same as in the vicinal Py/Cu(001) film but opposite to the vicinal Co/Cu(001) film. In addition, we notice that the shape of the hysteresis loop in vicinal Cu/Py/Ni/Cu(001) is different from that of vicinal Cu/Py/Cu(001) but similar to the hard axis loop of vicinal Cu/Co/Cu(001)^19^, showing that the 4-fold anisotropy can no longer be ignored in the vicinal Cu/Py/Ni/Cu(001) system. Then the magnetic anisotropy energy per unit area for the Py/Ni bilayer film will be


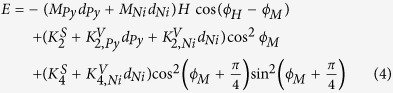


Here *K*^*S*^ and *K*^*V*^ denote surface (including all interfaces) and volume anisotropies, and the subscriptions of “2” and “4” denote the uniaxial and 4-fold anisotropies, respectively. The 4-fold anisotropy of Py film is ignored because it is too small. Minimizing eqn. (4) leads to the following equation.


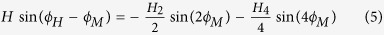


with
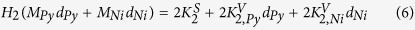



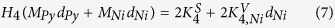


From eqn. (6), it is easy to see that at a fixed Ni thickness, *H*_*2*_(*M*_*Py*_*d*_*Py*_+*M*_*Ni*_*d*_*Ni*_) should depend on d_Py_ linearly with the linear slope being the Py volume uniaxial anisotropy and vice versa. ROTMOKE measurement was performed on vicinal Cu/Py/Ni/Cu(001) as a function of Py thickness at d_Ni_ = 4.8 ML and d_Ni_ = 9.0 ML. Figure 4(b) shows the result of the anisotropy field versus Py thickness. The same slope of the two straight lines in Fig. 4(b) shows that 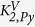 remains the same in vicinal Cu/Py/Ni/Cu(001) for Ni between 4.8 ML and 9 ML. Then we conclude that the Ni film consists a step-induced uniaxial anisotropy which favors the magnetization perpendicular to the steps, confirming the hysteresis loop measurement in Fig. 4(a). A linear fitting using eqn. (6) yields 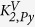 = (4.5 ± 0.2) × 10^4^ erg/cm^3^, where *M*_*Ni*_ = 490 Oe has been used. This value is greater than the value of 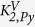 = 3.1 × 10^4^ erg/cm^3^ in vicinal Cu/Py/Cu(001), showing that the Ni underlayer in vicinal Cu/Py/Ni/Cu(001) changes the Py step-induced anisotropy. This is not surprising because Py grown on top of Ni/Cu(001) should be slightly different than Py grown on top of Cu(001) (e.g, strain due to lattice mismatch). The interesting observation is that 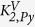 changes from the value in vicinal Cu/Py/Cu(001) to the value in vicinal Cu/Py/Ni/Cu(001) within 4.8 ML Ni film, suggesting that any structural changes in the Py film due to the Ni underlayer should have occurred within 5 ML Ni thickness. Alternatively, the Ni magnetic anisotropy could/should also undergo a change below and above ~5 ML thickness.

**Figure 5:**
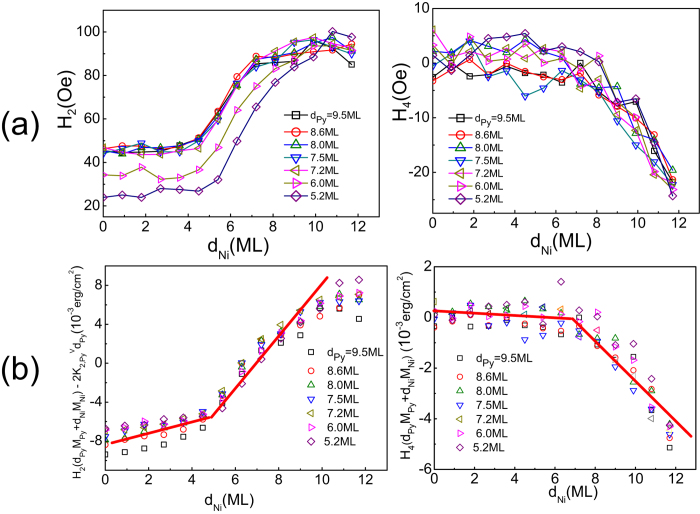
(**a**) Magnetic anisotropy fields of *H*_*2*_ and *H*_*4*_ from ROTMOKE versus Ni thickness for vicinal Cu/Py/Ni/Cu(001) at different Py thicknesses. (**b**) *H*_*2*_(*M*_*py*_*d*_*Py*_ + *M*_*Ni*_*d*_*Ni*_) − 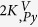 *d*_*Py*_and *H*_*4*_(*M*_*Py*_*d*_*Py*_ + *M*_*Ni*_*d*_*Ni*_) as a function of Ni thickness. All curves in (**a**) collapse into a universal curve in (**b**), showing the validity of eqn. (6) and (7). However, the Ni films clearly have different anisotropy constants below ~5 ML and above ~6 ML thickness.

To retrieve the Ni anisotropy in Py/Ni bilayers, we performed ROTMOKE measurement as a function of Ni thickness at different Py thicknesses in the range of 5 ML < d_Py_ < 9 ML where the 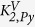 has a constant value of 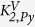 = 4.5 × 10^4^ erg/cm^3^. We fit the ROTMOKE data using eqn. (5) by considering both the uniaxial and 4-fold magnetic anisotropies in vicinal Cu/Py/Ni/Cu(001) films. Figure 5(a) shows the results of *H*_*2*_ and *H*_*4*_ from the ROTMOKE data fitting using eqn. (5). Both *H*_*2*_ and *H*_*4*_ increase with increasing Ni thickness. To single out the anisotropy constants of the Ni film from the data, we plot *H*_*2*_(*M*_*Py*_*d*_*Py*_ + *M*_*Ni*_*d*_*Ni*_)−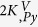 *d_Py_* and *H*_*4*_(*M*_*Py*_*d*_*Py*_ + *M*_*Ni*_*d*_*Ni*_) as a function of Ni thickness at different Py thicknesses [Fig. 5(b)]. According to eqn. (6) and (7), all curves should collapse into a single straight line with the slope being the volume anisotropy of the Ni film and the intersection of the straight line at d_Ni_ = 0 being the surface anisotropy of the Ni film. While Fig. 5(b) indeed shows a universal behavior for all curves, it also shows that the Ni magnetic anisotropies have different values for d_Ni_ < ~5 ML and d_Ni_ > ~6 ML.

From a linear fitting using eqn. (6) and (7) [red lines in Fig. 5(b)], we obtained the Ni anisotropy constants for d_Ni_ < 5 ML and d_Ni_ > 6 ML. The result is summarized in Table 1. We do not know the exact reason which causes the different Ni anisotropy constants below and above ~5–6 ML thickness. But this behavior is consistent with our observation that 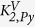 changes from 3.1 × 10^4^ erg/cm^3^ in vicinal Cu/Py/Cu(001) to 4.5 × 10^4^ erg/cm^3^ in vicinal Cu/Py/Ni/Cu(001) in the range of 0 < d_Ni_ < 4.8 ML. This behavior is also consistent with the result of Ref. 27 which shows that the Ni in-plane component behaves differently below and above ~5.5 ML thickness.

We would like to point out that the Ni magnetic anisotropy is very sensitive to volume strain and temperature^29^,^30^,^31^ as compared to Fe and Co. The different behaviors of the Ni anisotropy below and above ~5–6 ML thickness may be due to its structural strain variation as a function of its film thickness. Obviously, more experimental and theoretical works are needed to fully understand this behavior.

## Discussion

Table 1 shows the fitted anisotropy constants (including interface and volume contributions). In Cu/Py/Cu(001) system, the opposite sign of 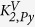 and 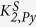 show that the interface and volume uniaxial anisotropies of the Py film prefer the easy magnetization axis being perpendicular and parallel to the steps, respectively. According to the general Néel’s expression for the effective anisotropy K_eff_ = K^V^ + 2 K^S^/d Ref. [32], the competition between the interface and the volume anisotropy energies is responsible for the overall effective uniaxial anisotropy induced by the steps and the interfacial contribution should gradually decrease with increasing the Py thickness.

Comparing the volume part of the step-induced uniaxial anisotropy, *K*^*V*^_*2,Ni*_ has a lower value than 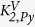 for Ni film thinner than 5 ML but a greater value than 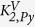 for Ni film thicker than 6 ML. For Ni film on vicinal Cu (001), it was reported that the Ni in-plane easy axis behave differently, resulting in a peculiar canted spin structure in the 5.5–7 ML thickness range^27^. Our result is consistent with the above behavior as the Ni step-induced magnetic anisotropy exhibits distinct different values below and above ~6 ML. This behavior is also evident in our 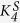 and 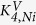 which also exhibit different values below and above ~6 ML Ni thickness. This phenomenon is different from the FeMn/Co/Cu(001) system although the antiferromagnetic FeMn layer induces a change of the Co four fold anisotropy^33^. It is should be mentioned that Ni film has a fct pseudomorphic growth on Cu(001) with an in-plane tensile strain because of the ~2.5% smaller lattice constant of bulk Ni than bulk Cu. A Ni film on Cu(001) will enlarge its in-plane lattice to that of Cu, leading to an out-of-plane contraction. Because of the special Ni electronic structure^34^, it is generally believed that a Ni lattice distortion in the out-of-plane direction should modify greatly the Ni magnetic anisotropy. LEED I-V study on Ni/Cu(001) has confirmed that the Ni film indeed contracts in the out-of-plane direction^35^ However, the experimental result could not further resolve a detailed thickness-dependent Ni lattice spacing at the instrumental limit of ~0.01-0.02 Å. If we trust the theoretical calculation^36^,^37^ a 0.01 Å change in the out-of-plane lattice spacing would result in a magnetic anisotropy change of ~20 μeV/atom which corresponds to a change of 6 × 10^−2^ erg/cm^2^ for surface anisotropy or a change of 3 × 10^6^ erg/cm^3^ for volume anisotropy. Both values are 10-100 times greater than the anisotropy change in Table 1. In another word, to account for the surface and volume anisotropy changes in Table 1 below and above 6 ML Ni, the out-of-plane lattice spacing has to be determined at the 0.001–0.0001 Å accuracy which is far beyond current experimental capability.

In summary, we investigated vicinal Cu/Py/Ni/Cu(001) with the atomic steps parallel to the [110] crystal axis. We found that atomic steps of the vicinal surface induce an in-plane uniaxial anisotropy in both Py and Ni films with the easy magnetization axis perpendicular to the steps. ROTMOKE was used to determine the step-induced magnetic anisotropy. By a systematic thickness-dependent study as a function of both Py and Ni thicknesses, we retrieved the anisotropy constants for Py and Ni films in vicinal Cu/Py/Ni/Cu(001). We show that Ni anisotropy behaves differently below and above ~5–6 ML thickness. Our result will provide valuable information for the control of in-plane step-induced magnetic anisotropy in bilayer systems grown on vicinal surfaces.

## Method

**Figure 6:**
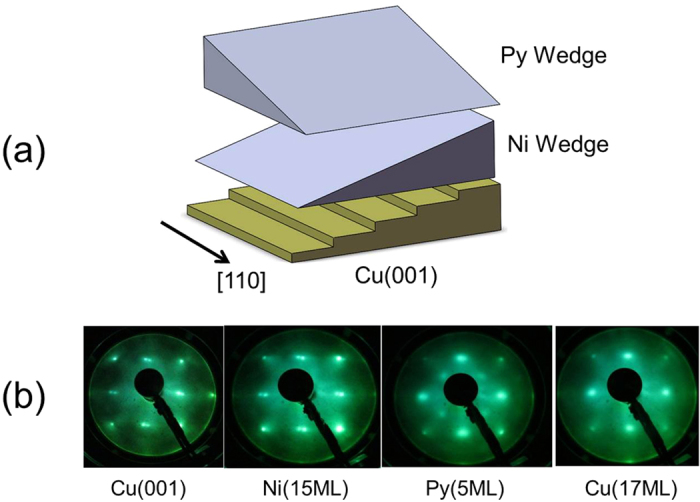
(**a**) Schematic drawing of the double wedged Py/Ni films on vicinal Cu(001). (**b**) LEED patterns taken at E~130 eV at each growth stage of the vicinal Cu/Py/Ni/Cu(001).

A 10 mm × 10 mm Cu(001) single crystal disk was polished into a 6^°^ vicinal surface with the steps parallel to the [110] crystalline axis. After cycles of cleaning in an ultrahigh vacuum (UHV) system by Ar ion sputtering at 2 keV and annealing at 600^°^C, sharp split low energy electron diffraction (LEED) spots were observed indicating the formation of a high quality vicinal Cu(001) surface (Fig. 6). Double wedges of Py and Ni films (Fig. 6) were grown epitaxially at room temperature in vacuum with a pressure below 3 × 10^−9^ torr. A 3 nm Cu layer was grown on top of the vicinal Py/Co/Cu(001) film to protect the sample from oxidation. The epitaxial growth nature of the Py/Ni films on top of the vicinal Cu(001) was confirmed by the LEED patterns (Fig. 6). Magneto-optical Kerr effect (MOKE) was used to measure the magnetic hysteresis loops of the sample with the magnetic field applied either perpendicular (polar MOKE) or parallel (longitudinal MOKE) to the sample surface. After the MOKE hysteresis loop measurement, the sample was taken out of the UHV chamber and measured using a table top rotation MOKE (ROTMOKE) setup. A detailed description of our ROTMOKE setup was reported previously^38^. All MOKE and ROTMOKE measurements were performed at room temperature.

## Additional Information

**How to cite this article**: Ma, S. *et al.* Tailoring the magnetic anisotropy of Py/Ni bilayer films using well aligned atomic steps on Cu(001). *Sci. Rep.* **5**, 11055; doi: 10.1038/srep11055 (2015).

## Figures and Tables

**Table 1:** Step-induced surface and volume uniaxial magnetic anisotropy in vicinal Cu/Py/Ni/Cu(001). 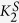 , 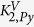 , 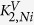 , 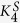 , and 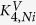 are defined in eqn. (4).

	**d_Ni_ < 5ML**	**d_Ni_ > 6ML**
*K*^*S*^_*2*_(10^−3^ erg/cm^2^)	−3.8 ± 0.1	−6.2 ± 0.3
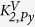 (10^4^ erg/cm^3^)	4.5 ± 0.2	4.5 ± 0.2
*K*^*V*^_*2,Ni*_(10^4^ erg/cm^3^)	1.3 ± 0.2	5.2 ± 0.2
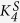 (10^−3^ erg/cm^2^)	<0.05	2.2 ± 0.2
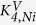 (10^4^ erg/cm^3^)	<0.09	−2.0 ± 0.1
